# LncRNA HULC enhances epithelial-mesenchymal transition to promote tumorigenesis and metastasis of hepatocellular carcinoma via the miR-200a-3p/ZEB1 signaling pathway

**DOI:** 10.18632/oncotarget.9883

**Published:** 2016-06-07

**Authors:** Shi-Peng Li, Hai-Xu Xu, Yao Yu, Jin-Dan He, Zhen Wang, Yan-Jie Xu, Chang-Ying Wang, Hai-Ming Zhang, Rong-Xin Zhang, Jian-Jun Zhang, Zhi Yao, Zhong-Yang Shen

**Affiliations:** ^1^ First Central Clinical College, Tianjin Medical University, Tianjin, P.R. China; ^2^ Oriental Organ Transplant Center of Tianjin First Central Hospital, Key Laboratory of Organ Transplantation of Tianjin, Tianjin, P.R.China; ^3^ Department of Immunology, Tianjin Key Laboratory of Cellular and Molecular Immunology, Key Laboratory of Immuno Microenvironment and Disease of the Educational Ministry, Tianjin Medical University, Tianjin, P.R.China; ^4^ Laboratory of Immunology and Inflammation, Department of Immunology, Key Laboratory of Immune Microenvironment and Diseases of Educational Ministry of China, Basic Medical College, Tianjin Medical University, Tianjin, P.R.China

**Keywords:** hepatocellular carcinoma, lncRNA HULC, epithelial-mesenchymal transition, miR-200a-3p, ZEB1

## Abstract

Highly upregulated in liver cancer (HULC), a lncRNA that is considered a key molecule in human liver cancer, has recently been revealed to be involved in hepatocellular carcinoma (HCC) development and progression [1, 2]. It has been reported that HULC can promote tumor invasion and metastasis of HCC, but its function and mechanism of action in HCC have not been elucidated. In this study, we found that HULC was aberrantly up-regulated in HCC tissues and associated with TNM stage, intrahepatic metastases, HCC recurrence, and postoperative survival. HULC depletion inhibited the growth and metastasis of HCC cell lines *in vitro* and *in vivo*. Moreover, HULC contributes to ZEB1-induced epithelial-mesenchymal transition (EMT), a requirement for tumor invasion and metastasis that plays a key role in cancer progression. This effect of ZEB1 was inhibited by HULC siRNA. We conclude that the HULC functioned as a competing endogenous RNA (ceRNA) to mediate EMT via up-regulating ZEB1. In this way, it sequesters the miR-200a-3p signaling pathway to facilitate HCC metastasis. HULC comes into play as an oncogene in HCC, acting mechanistically by inducing HCC cells to activate EMT. Such an effect promotes tumor progression and metastasis through the miR-200a-3p/ZEB1 signaling pathway. The identification of this novel pathway that links high expression levels of HULC with EMT in HCC cells may serve as the foundation for the development of novel anti-tumor therapeutics.

## INTRODUCTION

Hepatocellular carcinoma (HCC) is the fifth most common cancer worldwide and has a high mortality rate due to a lack of effective treatments. Despite advances in the diagnosis and management of HCC, the biology of this cancer remains largely unknown [3]. Recent evidence has indicated long noncoding RNAs (lncRNAs) as crucial determinants of HCC development. LncRNAs are defined as transcripts containing more than 200 nucleotides which act as decoys and guides to facilitate both proximal and distal macromolecular interactions [4–6]. More recent investigations have revealed that lncRNAs exert multiple functions upon a wide range of biological processes, including proliferation, apoptosis, cell migration, and cell invasion [7–9]. Of particular relevance to the present investigation, lncRNAs are dysregulated in different types of cancer and can exert critical effects as related to cancer biology.

One of the identified lncRNAs specifically, highly up-regulated in liver cancer (HULC), located on chromosome 6p24.3 and conserved in primates, is overexpressed in HCC [2]. Transcription of HULC yields an approximately 500 nt long, spliced and polyadenylated ncRNA that localizes to the cytoplasm, where it has been reported to be associated with ribosomes [10]. HULC was originally identified as being strongly overexpressed in noncoding transcripts of human HCC. At this site it is transactivated by CREB and sequesters miR-372 by acting as a sponge, and IGF2BP are able to govern the expression of HULC [11]. Hepatitis B virus X protein (HBx)-elevated HULC has been shown to accelerate the growth of hepatoma cells by downregulating p18 [12] and HULC increases abnormal lipid metabolism in HCC cells through an miR-9-mediated RXRA signaling pathway [13]. In addition to its influence in HCC, HULC also plays an important role in the growth and tumorigenesis of human gastric cancer (GC). With regard to GC, HULC can not only function as a new biomarker for GC, but also as a potential target for GC prevention, diagnosis and therapeutic treatment [14]. HULC may also serve as a prognostic biomarker candidate through its capacity for regulating growth in human pancreatic cancer [15]. However, to date, there exists no direct evidence regarding the mechanisms through which HULC mediates tumor cell invasion and metastasis.

Increasing evidence has been presented which suggests that the epithelial-mesenchymal transition (EMT) contributes to tumor metastasis and recurrence, including that involving HCC. As a result, research involving EMT has become one of the most exciting areas of investigation in cancer biology. While its role in cancer cell invasion, metastasis and drug resistance is well established, the molecular basis of EMT remains unknown [16]. An increasing number of transcription factors, which can potentiate EMT, including Twist, ZEB1 and Snail have been identified [17]; and recently, lncRNA H19 was characterized as a novel regulator of EMT in colorectal cancer. H19, as a competing endogenous RNA (ceRNA) for miR-138 and miR-200a, antagonized the functions of these miRNAs which results in the de-repression of their endogenous targets, Vimentin, ZEB1 and ZEB2. The ceRNA hypothesis proposes that a large number of non-coding RNAs might function as molecular sponges for miRNAs and hence functionally liberate other RNA transcripts targeted by aforementioned active miRNAs [18]. One interpretation of these observations is that the lncRNA H19 modulates the expression of multiple genes involved in EMT by acting as a ceRNA to construct the missing link between the regulatory miRNA network and EMT progression [19]. Related to this topic are the findings that lncRNA-ATB plays an important role with regard to EMT's capacity to promote invasion and metastasis through the TGF-β1/miR-200s/ZEB axis, resulting in a poor prognosis in gastric cancer patients [20].

In the present study, we found the expression of HULC to be significantly up-regulated in HCC tissues as compared with that in normal tissues. This enhanced expression of HULC was correlated with poor patient prognosis. Further, as based upon results obtained from *in vitro* functional assays, increased HULC expression significantly increased cell proliferation. Whether HULC participates in a similar processes involving EMT in HCC cells is unclear. To address this issue, we investigated the relationship between HULC expression and EMT in HCC cells. Our working hypothesis was that HULC functioned as a ceRNA and promoted EMT via up-regulating ZEB1. Such an effect would result in the sequestering of the miR-200a-3p signaling pathway thereby contributing to tumor growth and metastasis. In this way, HULC could promote HCC migration and invasiveness by enhancing EMT. To complement these *in vitro* experiments, an animal model, was included to confirm that HULC contributed to promoting tumor growth and metastasis as examined under *in vivo* conditions.

## RESULTS

### HULC is up-regulated in HCC tissues

**Figure 1:**
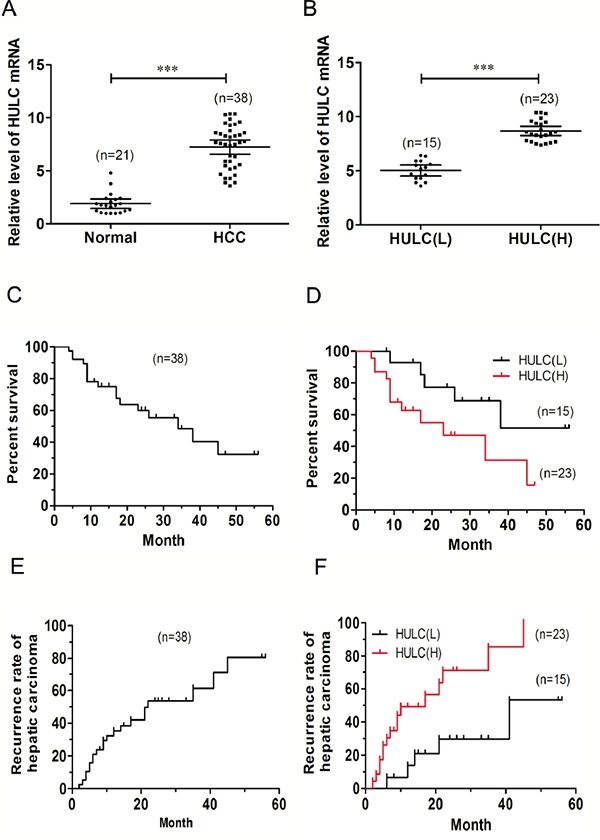
Relative HULC expression in HCC tissues and its relationship with overall survival of HCC patients **A.** Relative expression of HULC in HCC tissues (n=38) compared with that of corresponding normal tissues (n=21). HULC expression as determined using qRT-PCR and normalized to GAPDH expression. **B.** HULC expression was classified into two groups HULC(L) and HULC(H). Kaplan-Meier survival curves and log-rank tests were used to evaluate survival in all patients **C.** and each of the two sub-groups (L versus H HULC) patients **D.** Kaplan-Meier survival curves and log-rank tests were used to evaluate recurrence rates in all patients **E.** and each of the two sub-groups of patients **F.** Values shown indicate mean ± 95% CI. ***P< 0.001.

QRT-PCR analysis was used to determine expression levels of HULC in HCC tissues (n=38) and normal liver tissues (n=21). We found that HULC was over-expressed in HCC tissues and these levels of HULC expression were significantly higher than that observed in normal liver tissues (P< 0.001, Figure 1A).

### HULC is associated with HCC progression

These changes in HULC expression were further evaluated within clinicopathologic samples. For this analysis, the relative HULC expression in tumor tissues from the 38 HCC patients were classified into two groups: HULC(L) - Low relative levels of HULC mRNA (n=15, fold change < 7.0) and HULC(H) - High relative levels of HULC mRNA (n=23, fold change ≥ 7.0, Figure 1B). Results of this clinicopathological analysis revealed that HULC was significantly correlated with clinical stage (TNM, P< 0.05) and intrahepatic metastases (P< 0.05). No statistically significant correlations were obtained between HULC and other clinicopathological characteristics such as age, gender, tumor size, tumor differentiation or distant metastasis (P> 0.05, Table 1).

**Table 1:** Association of lncRNA HULC expression with clinicopathologic features in HCC patients

Parameter	Total	LncRNA HULC		*χ^2^*	P
		Low	High		
Age (years)					
<60	23	10	13	0.391	0.532
≥60	15	5	10		
Gender					
Female	9	4	5	-	1.000 ^*^
Male	29	11	18		
Tumor size (cm)					
<5	15	5	10	0.391	0.532
≥5	23	10	13		
Tumor differentiation					
Well/moderate	22	11	11	2.423	0.120
Poor	16	4	12		
Clinical stage (TNM)					
I~II	23	12	11	3.934	0.047
III~IV	15	3	12		
Intrahepatic metastases					
No	17	10	7	4.821	0.028
Yes	21	5	16		
Distant metastasis					
No	28	14	15	-	0.061*
Yes	10	1	8		
HCC recurrence					
No	17	10	7	4.821	0.028
Yes	21	5	16		

* Fisher exact test

The Kaplan-Meier analysis and log-rank test were used to evaluate the relationship between HULC expression in HCC and patient survival. The median survival of all HCC patients was 34 months (Figure 1C). A high HULC expression was significantly associated with a poor 5-year overall survival rate in HCC patients; and the median survival of the high HULC expression group (17 months) significantly decreased (P<0.05) as compared with that of the low HULC expression group (41 months, Figure 1D). These results showed that the recurrence rate of HCC in patients with high levels HULC expression was 69.61% versus 50.0% for those with low HULC expression levels (P<0.05, Table 1). Median recurrence of all HCC patients was 22 months (Figure 1E), and median recurrence of the high HULC expression group was shorter than that of the low HULC expression group (P<0.05, Figure 1F). Collectively, these results suggest that the up-regulation of HULC may be involved in development, progression and prognosis of HCC within the majority of patients with this condition.

### Knockdown of HULC inhibits HCC cell proliferation and induce apoptosis *in vitro*

**Figure 2:**
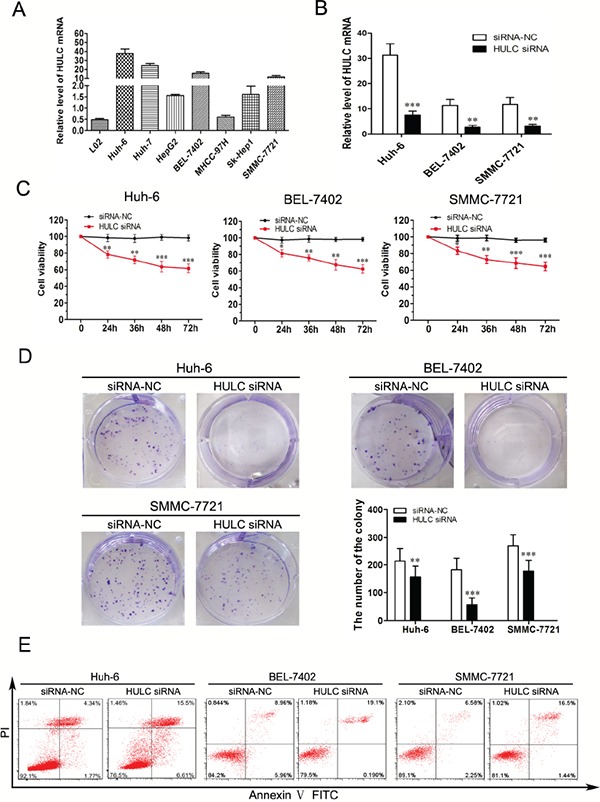
Effect of HULC on HCC cell growth *in vitro* **A.** Analysis of HULC expression levels in HCC cell lines (Huh-6, Huh-7, HepG2, BEL-7402, MHCC-97H, Sk-Hep1 and SMMC-7721) compared with L02 cell as determined using qRT-PCR and normalized to GAPDH expression. **B.** Relative expression levels of HULC in Huh-6, BEL-7402 and SMMC-7721 cells transfected with siRNA-NC or HULC siRNA, as determined using qRT-PCR. **C.** CCK8 was performed to determine cell viability and proliferation of Huh-6, BEL-7402 and SMMC-7721 cells. **D.** Representative results of colony formation of Huh-6, BEL-7402 and SMMC-7721 cells transfected with siRNA-NC or HULC siRNA. **E.** Huh-6, BEL-7402 and SMMC-7721 cells were stained and analyzed by flow cytometry after transfection for 24 hours. Values shown indicate mean ± SD. *P < 0.05, **P < 0.01, ***P < 0.001.

As shown in Figure 2A, seven cell lines (Huh-6, Huh-7, HepG2, BEL-7402, MHCC-97H, Sk-Hep1 and SMMC-7721) expressed high levels of HULC compared with the normal human hepatic cell line (L02). The over-expressing HULC cell lines, Huh-6, BEL-7402 and SMMC-7721, were selected for further analysis. HULC siRNA was then transfected into these three cell lines and HULC expression was significantly reduced in Huh-6, BEL-7402 and SMMC-7721 cell lines (Figure 2B). The CCK-8 assay was used to assess the effects of knockdown of HULC expression. The results of this assay revealed that cell viability and proliferation in Huh-6, BEL-7402 and SMMC-7721 cell lines were significantly inhibited as compared with the siRNA-NC (Figure 2C). The survival ratios were compared between groups and time points in each cell line, following a GLM univariate procedure of SPSS 19.0. The effects of group, time and group*time all reached statistical significance in the Huh-6, BEL-7402 and SMMC-7721 cell lines. There was a significant difference in the trend of the survival curves between the two groups in each cell line (P<0.001, P<0.001, P<0.001). Then the comparisons of cell survival ratios between HULC siRNA and siRNA-NC group, were then conducted at each time point respectively (Figure 2C). Significant differences between groups were found at the time points (24 h, 36 h, 48 h and 72 h, Figure 2C). Similarly, results of the colony-formation assay revealed that clonogenic survival was significantly decreased following inhibition of HULC in Huh-6, BEL-7402 and SMMC-7721 cell lines (Figure 2D). Next, flow cytometric analysis was performed to further examine whether this effect of HULC on HCC cells proliferation involved an alteration in apoptosis. The results of this assay demonstrated that HULC knockdown was clearly involved in inducing cell apoptosis (Figure 2E).

### HULC increased ZEB1 by sequestering miR-200a-3p

**Figure 3:**
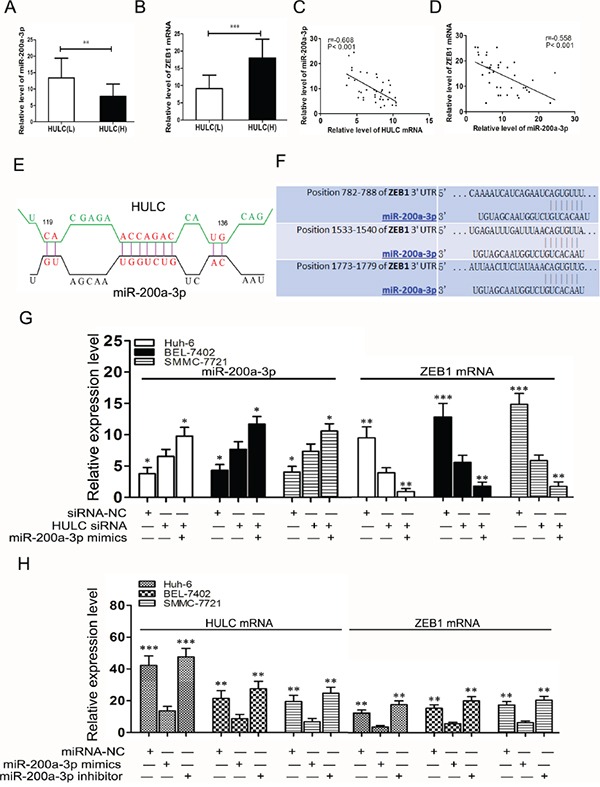
HULC increased ZEB1 by sequestering miR-200a-3p **A.** Analysis of miR-200a-3p expression levels in HULC(L) and HULC(H) groups of HCC tissues as determined using qRT-PCR and normalized to GAPDH expression. **B.** QRT-PCR was used to detect expression levels of ZEB1 mRNA in HULC(L) and HULC(H) groups of HCC tissues and normalized to GAPDH expression. **C.** Correlations between HULC and miR-200a-3p expression in HCC tissues. **D.** Correlations between miR-200a-3p and ZEB1 mRNA expression in HCC tissues. **E.** A model showing the predicted interaction between HULC and miR-200a-3p through complementary base-pairs. **F.** A photograph showing the predicted interaction between miR-200a-3p and ZEB1 mRNA through complementary base-pairs.**G.** Relative expressions of miR-200a-3p and ZEB1 mRNA as determined using qRT-PCR in Huh-6, BEL-7402 and SMMC-7721 cells transfected with HULC siRNA or miR-200a-3p mimics. **H.** Relative expressions of ZEB1 and HULC mRNA were measured by qRT-PCR in Huh-6 and BEL-7402 cells transfected with miR-200a-3p mimics or inhibitor. Values shown indicate mean ± SD. *P < 0.05, **P < 0.01, ***P < 0.001.

To determine whether HULC regulates EMT by affecting miR-200a targets, we first evaluated the effect of miR-200a-3p on the ZEB1 target. Findings from a previous study exhibited various levels of metastatic potential that are mainly regulated by ZEB1 and miR-200-3p [21]. ZEB1 and miR-200-3p form a double-negative feedback loop that plays a key role in determining the metastatic fate of epithelial cancers through the regulation of downstream target genes and miRNAs [22]. When comparing expression levels of miR-200a-3p between HULC(L) and HULC(H) tissues, we found these levels to be significantly lower (P<0.01, Figure 3A). However, an opposite result was obtained when measuring ZEB1 mRNA (P<0.001, Figure 3B). Within our 38 clinical HCC tissues samples, we found that expression levels of HULC were negatively correlated with those of miR-200a-3p (r=−0.608, P<0.001, Figure 3C). Meanwhile, the expression levels of miR-200a-3p were negatively correlated with those of ZEB1 mRNA (r=−0.558, P<0.001, Figure 3D).

With use of alignment prediction as a means to investigate the molecular mechanisms of lncRNA HULC in HCC cells, we found that HULC was aligned with sequences of miR-200a-3p (Figure 3E). According to the bio-informatics database (http://www.targetscan.org/, http://zmf.umm.uni-heidelberg.de/apps/zmf/mirwalk2/), we predicted that the miR-200a-3p binding site was at the 3′-UTR of ZEB1 (Figure 3F). This prediction was similar to that presented in a previous report indicating that ZEB1 was a target of miR-200a-3p as determined via a luciferase reporter [19]. When the expression of HULC in HCC cells transfected by HULC siRNA was inhibited, a significant increase in miR-200a-3p expression was observed as compared with siRNA-NC (P<0.05), and miR-200a-3p mimics could further promote miR-200a-3p expression. In contrast, the expression of ZEB1 mRNA significantly decreased (P<0.01, Figure 3G). Moreover, HULC and ZEB1 mRNA levels decreased after transfection with miR-200a-3p mimics compared with that of miR-NC (P<0.01). However, these HULC and ZEB1 mRNA levels increased after transfection with the miR-200a-3p inhibitor as compared with that of miR-NC (P<0.05, Figure 3H). Taken together, these data indicate that miR-200a-3p was negatively regulated by HULC in HCC cells, and HULC functioned as a ceRNA to up-regulate ZEB1 by sequestering miR-200a-3p.

### HULC expression level was associated with EMT features of HCC

**Figure 4:**
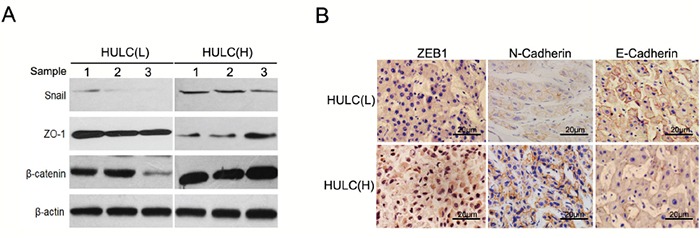
HULC was associated with EMT features of HCC **A.** Western blot was used to detect expression of Snail, ZO-1and β-catenin in HULC(L) and HULC(H) groups as normalized to β-actin expression. **B.** Immunohistochemisty revealed expression of ZEB1 in the cytoplasm and nucleus of HCC cells. Expression of N-Cadherin and E-Cadherin in the plasmalemma and cytoplasm of HCC cells (magnification, 200×, scale bars=20 μm).

As we found that high levels of HULC expression were strongly associated with increasing metastasis in HCC, we used western blot (Figure 4A) and immunohistochemistry (Figure 4B) to analyze EMT markers, including E-Cadherin, N-Cadherin, ZO-1, Vimentin, β-Catenin, Snail and ZEB1, to compare EMT features between HULC(L) and HULC(H) within our clinical HCC samples. Our data suggested that a positive correlation existed between the expression of HULC and some EMT features, which likely contribute to the observed aggravation of tumor metastasis in HCC.

### HULC promoted epithelial to mesenchymal transition *in vitro*

**Figure 5:**
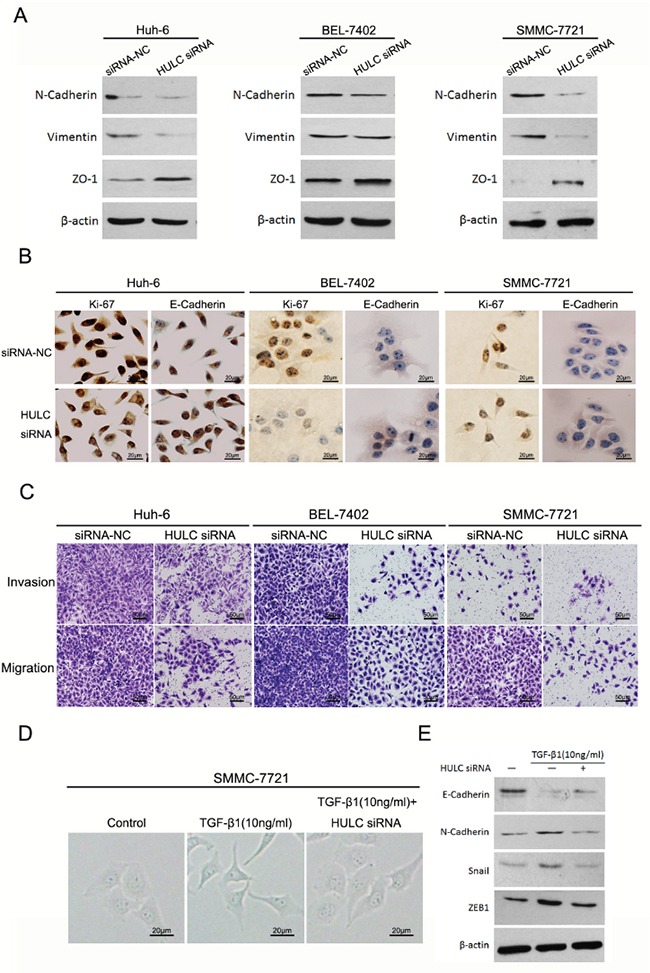
HULC promoted epithelial to mesenchymal transition *in vitro* **A.** Western blot was used to measure expression levels of EMT markers (N-Cadherin, Vimentin and ZO-1) in Huh-6, BEL-7402 and SMMC-7721 cells transfected with HULC siRNA or siRNA-NC, and normalized to β-actin expression. **B.** Expression levels and location of Ki-67 and E-Cadherin in Huh-6, BEL-7402 and SMMC-7721 transfected with siRNA-NC or HULC siRNA as determined using immunocytochemistry (magnification, 200×, scale bars=20 μm). **C.** The effect of decreasing HULC expression on cell invasion and migration of Huh-6, BEL-7402 and SMMC-7721 cells as assessed using a transwell assay (magnification, 100×, scale bars=50 μm). **D.** The effect of HULC on morphological differences in SMMC-7721 cells treated with TGF-β1 (magnification, 400×, scale bars=20 μm). **E.** Measures of expression levels of EMT markers (E-Cadherin, N-Cadherin, Snail and ZEB1) as determined using western blot in SMMC-7721 cells treated with TGF-β1 or transfected with HULC siRNA, and normalized to β-actin expression.

To confirm the function of lncRNA HULC in HCC EMT, we examined the expressions of N-Cadherin and Vimentin in response to HULC. All were significantly down-regulated in Huh-6, BEL-7402 and SMMC-7721 cells by HULC siRNA. However, HULC inhibition restored ZO-1 expression (Figure 5A). HULC inhibition increased E-Cadherin expression in HCC cells (Figure 5B). The expression and activity of Ki-67 was inhibited when HCC cells were treated with HULC siRNA (Figure 5B). As shown in Figure 5C, we found that HULC siRNA respectively decreased capabilities of invasion and migration in HCC cells. These results clearly provide new insights into the mechanisms through which HULC can act as a tumor promoter to enhance the progression of HCC.

Reportedly, EMT can be induced by TGF-β1 within the tumor microenvironment [23]. To test whether HULC is involved in this TGF-β1-induced EMT, SMMC-7721 cells were treated with TGF-β1 (10 ng/ml) for 5d. As shown in Figure 5D, the treatment of TGF-β1 produced an alteration in the shape of SMMC-7721 cells from an epithelial-like to spindle-like profile, however, HULC siRNA attenuated SMMC-7721 cell shape. In addition, we found that TGF-β1 produced a significant decrease in E-Cadherin expression, but increased expressions of N-Cadherin, Snail and ZEB1 in SMMC-7721 cells. However, a decrease in the expression of HULC prevented this decrease in E-Cadherin expression and increased N-Cadherin, Snail and ZEB1 in SMMC-7721 cells treated with TGF-β1 in Figure 5E.

### HULC enhanced epithelial to mesenchymal transition by sequestering miR-200a-3p in HCC cells

**Figure 6:**
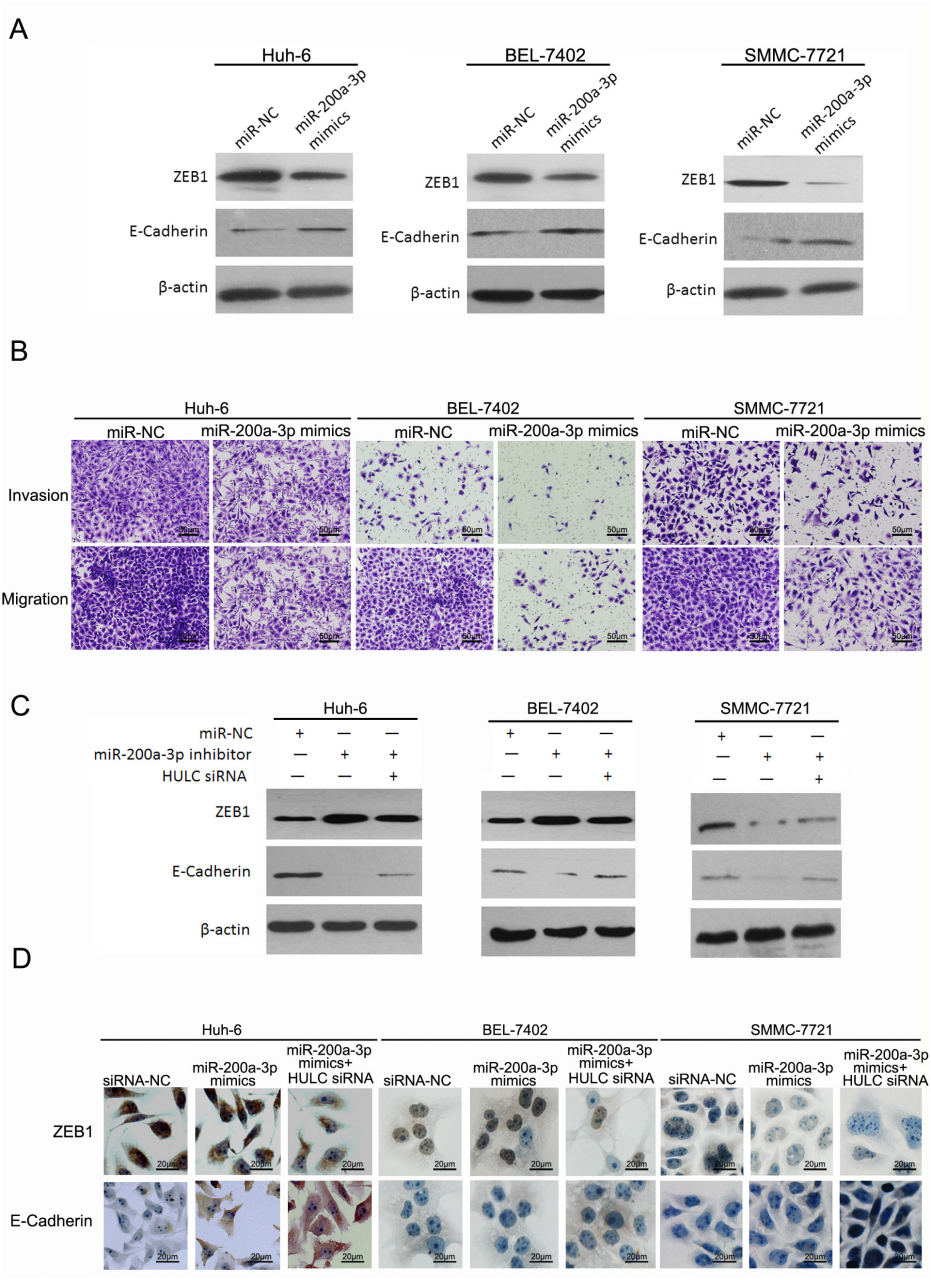
HULC enhanced epithelial to mesenchymal transition by sequestering miR-200a in HCC cells **A.** Western blot was used to measure expression levels of ZEB1 and E-Cadherin in Huh-6, BEL-7402 and SMMC-7721 cells transfected with miR-200a-3p mimics or miR-NC, and normalized to β-actin expression. **B.** The effect of increasing miR-200a-3p expression in cell invasion and migration of Huh-6, BEL-7402 and SMMC-7721, transfected with miR-200a-3p mimics or miR-NC, as determined using a transwell assay (magnification, 100×, scale bars=50 μm). **C.** Expression levels of ZEB1 and E-Cadherin as determined using western blot in Huh-6, BEL-7402 and SMMC-7721 cells transfected with the miR-200a-3p inhibitor, miR-NC or HULC siRNA, and normalized to β-actin expression. **D.** Expression levels and location of ZEB1 and E-Cadherin as determined using immunocytochemistry in Huh-6, BEL-7402 and SMMC-7721 cells transfected with miR-200a-3p mimics, miR-NC or HULC siRNA (magnification, 400×, scale bars=20 μm).

To verify whether HULC enhanced EMT by sequestering miR-200a-3p in HCC cells, we measured protein levels of ZEB1 within miR-200a-3p in HCC cells. As expected, restored expression of miR-200a-3p decreased the protein levels of ZEB1 in Huh-6, BEL-7402 and SMMC-7721 cells treated with miR-200a-3p mimics. Protein levels of E-Cadherin increased significantly in HCC cells treated with miR-200a-3p mimics (Figure 6A). While up-regulating miR-200a expression decreased the invasion and migration capabilities of HCC cells (Figure 6B), inhibiting the expression of miR-200a-3p increased the levels of ZEB1 but decreased the levels of E-Cadherin in HCC cells treated with miR-200a-3p inhibitor (Figure 6C). However, HULC siRNA inhibited these changes in ZEB1 and E-Cadherin expression in HCC cells treated with the miR-200a-3p inhibitor (Figure 6C). As determined with immunocytochemistry, both intranuclear and intracytoplasmic levels of ZEB1 decreased, but E-Cadherin expression increased in HCC cells treated with miR-200a-3p mimics. In addition, HULC siRNA promoted changes in ZEB1 and E-Cadherin expression in HCC cells treated with miR-200a-3p mimics (Figure 6D). These results suggest that HULC can interfere with miR-200a-mediated inhibition of ZEB1, leading to differentiation from an epithelial to mesenchymal transition.

### HULC promoted tumor growth and intrahepatic metastasis *in vivo*

**Figure 7:**
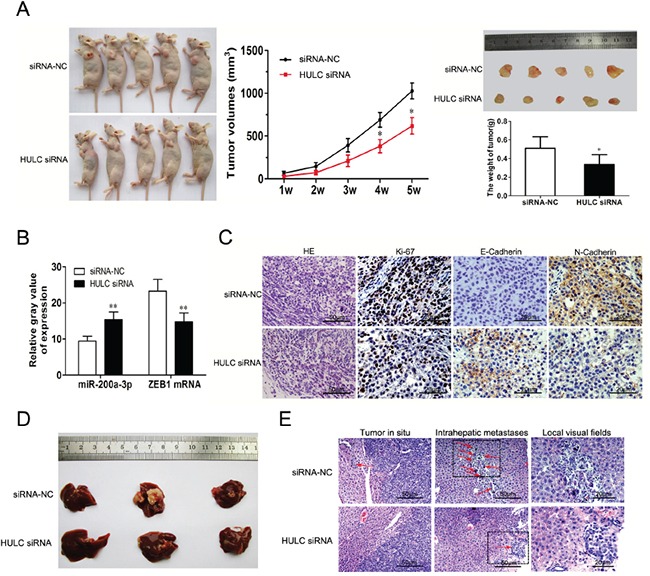
The impact of HULC on tumorigenesis and intrahepatic metastases *in vivo* **A.** LV-siRNA-NC or LV-HULC siRNA was transfected into Huh-6 cells, which were injected into nude mice (n=5). Tumor volumes were calculated after injection at weekly intervals. **B.** QRT-PCR was performed to detect the average expression of miR-200a-3p and ZEB1 mRNA in xenograft tumors (n=5). **C.** Ttumor sections under H&E staining (magnification, 100×, scale bars=50 μm) and IHC staining (magnification, 200×, scale bars=20 μm) to detect Ki-67, E-Cadherin and N-Cadherin. **D.** and **E.** Representative images of intrahepatic metastases (red arrows) based on H&E staining (magnification, 100×/200×, scale bars=50/20 μm) in nude mice 5 weeks after liver injection with SMMC-7721 cells that were transfected with LV-siRNA-NC or LV-HULC siRNA. Values shown indicate means ±SD,*P < 0.05, **P < 0.01.

Tumors from the LV-HULC siRNA transfected Huh-6 cells grew at a slower rate than that of those transfected with LV-siRNA-NC alone. As compared with siRNA-NC group, HULC siRNA transfected Huh-6 cells displayed a significant decrease in tumor size and weight (Figure 7A). By the result of a GLM univariate analysis, the effects of group, time and group-time reached statistical significant (P<0.001, P<0.001, P<0.001). Thus a comparison of tumor size between HULC siRNA and siRNA-NC group, was conducted at each time point respectively (Figure 7A). There were significant differences in the tumor sizes in week 4 and week 5 (P<0.05, P<0.05). Compared with siRNA-NC group, there was a high-expression of miR-200a-3p, but a low-expression of ZEB1 mRNA in HULC siRNA tumor samples (P<0.01) from the nude mice (Figure 7B). Immunohistological staining showed that the expression of E-Cadherin was upregulated in HULC siRNA tumor samples from nude mice as compared with siRNA-NC alone samples. The expression of N-Cadherin showed an opposite trend. Furthermore, expression of the cell proliferation marker Ki-67, which was used for evaluating *in vivo* tumor growth, was inhibited in HULC siRNA tumor tissues (Figure 7C). Metastatic liver nodules from the LV-HULC siRNA or LV-siRNA-NC transfected SMMC-7721 cells were visible on the liver surface in the siRNA-NC group (Figure 7D). Strikingly, intrahepatic metastases were substantially fewer in the HULC siRNA group than in siRNA-NC (Figure 7E).

## DISCUSSION

An increasing number of studies have indicated that lncRNAs are implicated in a variety of cellular processes and critical functions within cancer biology [24–26]. With regard to these latter effects, expression levels of certain lncRNAs have been shown to be associated with the recurrence, metastasis and prognosis of various cancers (HCC, gastric carcinoma, breast carcinoma) [27–29]. Mounting evidence indicates that HULC plays an essential role in liver tumor development, and HULC, as an oncogenic factor in human tumor progression, is upregulated in HCC [1]. Previous studies reported HULC was a negative prognostic factor for liver cancer [2], and our clinical data showed similar results that HULC expression in HCC tissues was significantly increased as compared with that observed in normal liver tissues. Moreover, HULC expression in HCC tissues was significantly correlated with clinical stage in HCC, as based upon clinicopathological analysis. However, we found HULC over-expression was closely correlated to intrahepatic metastasis. Analyses of the relationship between high versus low levels of HULC expression in HCC patient survival, as performed using the Kaplan-Meier analysis and logrank test, indicated that overexpression of HULC may be involved in the development, progression and prognosis of HCC in the majority of patients with this condition. These results are in accord with other findings which show that HULC is correlated with tumor size, lymph node metastasis and vascular invasion in pancreatic [15] and gastric cancers [14].

Although increased levels of HULC expression have been implicated in tumor invasion and metastasis, the exact mechanisms and downstream mediators of these effects remain to be elucidated. Epithelial-mesenchymal transition (EMT), a morphogenetic process that results in a loss of epithelial characteristics and acquisition of a mesenchymal phenotype, has become one of the most exciting and increasingly investigated areas in cancer biology [30–32]. Emerging evidence suggests that EMT plays a key role in cancer progression [33–35]. Previous studies showed that silencing of HULC effectively reversed EMT phenotype, which provided us with a new biomarker in GC and perhaps a potential target for GC [14]. HULC, as a Sp-regulated gene, was important for liver cancer cell growth, migration, invasion and EMT. Thus, drugs such as metformin that downregulate Sp transcription factors and HULC may be clinically useful in drug combinations for treating HCC patients [36].

Zinc finger E-box-binding homeobox (ZEB) 1 and E-box-binding transcription factors have been reported to be involved in tumorigenesis of various malignancies [9]. ZEB1 is an important modulator in the network of transcriptional repressors that suppress many pivotal regulators of epithelial polarity and, in this way, are critically involved with EMT [37, 38]. Results from previous studies have shown that ZEB1 plays an important role in regulating E-Cadherin expression in tumor invasion and metastasis [39], and its expression is closely related to the prognosis of cancer [40, 41].

In the present study, we found that the expression of miR-200a-3p was increased in HULC(L) HCC tissues (Figure 3A), while ZEB1 mRNA expression was decreased (Figure 3B). Expression levels of HULC were negatively correlated with those of miR-200a-3p in HCC samples (Figure 3C). And, in turn, miR-200a-3p expression was negatively correlated with that of ZEB1 mRNA in HCC samples. Therefore, we hypothesized that HULC might promote angiogenesis as related to changes in ZEB1 and miR-200a-3p. It has been reported that ZEB1 is a target of miR-200a-3p via a luciferase reporter [42]. Notably, the EMT activators transforming growth factor beta2 and ZEB1 are the predominant targets downregulated by miR-200c. These results indicate that ZEB1 triggers an miRNA-mediated feedforward loop that stabilizes EMT and promotes invasion of cancer cells [43]. Loss of expression of the miR-200 family members may play a critical role in the repression of E-cadherin by ZEB1 and ZEB2 during EMT, thereby enhancing migration and invasion during cancer progression [44]. miR-200 suppression in poor prognosis of some colorectal cancer (CRC) cells may promote ZEB1-mediated cancer metastasis [45].

Given that the ceRNA hypothesis, which proposes that a large number of non-coding RNA might function as molecular sponges for miRNAs and hence functionally liberate other RNA transcripts targeted by active miRNAs [46], we hypothesized that lncRNA HULC might serve as a ceRNA by sequestering miR-200a during EMT progress. Based upon the bio-informatics database, we performed alignment prediction and found that HULC was aligned with sequences of miR-200a-3p (Figure 3E) and subsequently verified the presence of a targeting relationship between HULC and miR-200a-3p. Accordingly, we present clear evidence indicating that miR-200a-3p was negatively regulated by HULC in HCC cells, and HULC functioned as a ceRNA to up-regulate ZEB1 by sequestering miR-200a-3p (Figure 3G).

The high levels of HULC expression which contribute to tumor invasion and metastasis might be related to EMT. When EMT markers from liver samples of HCC patients were analyzed, we found high expressions of HULC which were associated with an increase in EMT-related parameters (Figure 4). These high levels of HULC expression promoted EMT as demonstrated *in vitro* (Figure 5). To verify whether HULC enhanced EMT by sequestering miR-200a-3p in HCC cells, we assessed the protein levels of ZEB1 in miR-200a-3p over- or under-expressing HCC cells. As expected, miR-200a-3p decreased protein levels of ZEB1 in HCC cells (Figure 6A). miR-200a-3p appears to act as a multifunctional tumor suppressor miRNA in meningioma, which might be related to ZEB1 and E-Cadherin [47, 48]. The E-Cadherin signal was decreased and ZEB1 increased following miR-200a knockdown. As a result, decreasing cavity formation rate and suppressing claudin-3 and par-6b expression to reduce epithelial cell polarity [49]. We also treated HCC cells with miR-200a-3p mimics/inhibitor and HULC siRNA to investigate the potential for interactions between miR-200a and HULC during EMT in HCC (Figure 6C). Meanwhile, HULC promoted tumor growth and intrahepatic metastasis *in vivo* (Figure 7). These results suggest that HULC can interfere with miR-200a-mediated inhibition of ZEB1, leading to a differentiation from EMT in HCC.

In aggregate, lncRNA HULC is a significant component of liver cancer cell growth, survival, invasion and migration. Here, we demonstrate that HULC enhanced epithelial-mesenchymal transition by sequestering miR-200a-3p in HCC cells. These findings provide a novel insight into HULC, as a lncRNA capable of enhancing EMT in HCC.

## MATERIALS AND METHODS

### Patient specimens

HCC tissues (n=38) and normal liver tissues (n=21) were obtained from the Tianjin First Center Hospital from 2008 to 2010. None of the patients received preoperative radiotherapy or chemotherapy prior to surgical resection. Clinical and pathological characteristics were obtained from patient charts. The histological diagnosis and differentiation were evaluated independently by three pathologists according to the WHO classification system. The clinicopathological features are summarized in Table 1. Tumor and normal liver specimens were snap-frozen in liquid nitrogen and stored at −80°C immediately after resection. This study was approved by the Ethics Committee of Tianjin First Center Hospital, and all patients provided written informed consent for the use of the tumor tissues for clinical research.

### Cell lines, siRNAs and antibodies

Seven human liver cancer cell lines (Huh-6, Huh-7, HepG2, BEL-7402, MHCC-97H, Sk-Hep1 and SMMC-7721) and a normal liver cell line (L02) were purchased from the Institute of Biochemistry and Cell Biology of the Chinese Academy of Sciences (Shanghai, China). Cells were maintained in Dulbecco's modified Eagle's medium (DMEM) with 10% fetal bovine serum(Thermo Scientific HyClone, Beijing, China), 100 U/ml penicillin and 100 mg/ml streptomycin in humidified air at 37°C with 5% CO_2_. LncRNA HULC Smart Silencer, miR-200a-3p mimics/inhibitor, miRNA negative control (miR-NC), and RiboFECTTM CP Reagent were purchased from RiboBio Co., Ltd. (Guangzhou, China). Lipofectamine 2000 was obtained from Invitrogen (Rockville, MD) and SYBR Green qRT-PCR Master Mix was purchased from Roche Diagnostics GmbH (Mannheim, Germany). Antibody ZEB1, E-Cadherin, N-Cadherin, Vimentin, Snail, β-Catenin, ZO-1, Ki-67, β-actin and the HRP-conjugated secondary antibody were purchased from Cell Signaling Technology (Inc. USA). All other reagents were purchased from Sigma or as indicated.

### Lentivirus vectors for HULC siRNA

To further investigate the function of HULC, HULC expression was modified by gene knockdown via lentivirus vector. For knockdown, LV-HULC siRNA (target sequence [14]: 5′-GCCTTTACAAGGGAATGAAGA-3′), with ≥75% knockdown efficiency, was used for further studies. All lentiviral vectors expressed GFP and the efficiency of infection was measured under a fluorescent microscope based on GFP expression.

### SiRNA transfection

Huh-6, BEL-7402 and SMMC-7721 cells (2×10^5^) were seeded into each well of 6-well plates and incubated for 24h. Cells were then transfected with 100 nmol/L of LncRNA HULC siRNA, miR-200a-3p mimics/inhibitor or a miR-NC that consisted of Lipofectamine 2000 transfection reagent.

### CCK-8 assay cell growth viability

After transfection, Huh-6, BEL-7402 and SMMC-7721 cells at a concentration of 5×10^3^ per well were seeded in the 96-well plate and incubated for 24, 48 or 72 h. Cell growth viability was measured with a CCK-8 (Beyotime, Shanghai, China), following the manufacturer's instructions. Absorbance (A) was then recorded at 450 nm using an Elx800 Reader (Bio-Tek Instruments Inc., Winooski, VT, USA).

### HCC EMT cell model

SMMC-7721 cells were seeded on 100-mm dishes and allowed to attach. Following attachment, the medium was changed to DMEM containing 1% FBS with or without TGF-β1 (10 ng/mL, PeproTech, USA) and the culture was continued for an additional of 5d [50].

### Colony formation assay

The transfected Huh-6, BEL-7402 and SMMC-7721 cells (800 cells/well) were placed in a fresh six-well plate. After 24 h, the medium was replaced with new medium. After 14 days, cells were fixed with methanol and stained with 0.1% crystal violet. Visible colonies were manually counted.

### Migration and invasion assays

For transwell migration assays, transfected Huh-6, BEL-7402 and SMMC7721 cells (4×10^5^) were plated within the top chamber with the non-coated membrane (BD Biosciences, San Jose, CA, USA). For invasion assays, matrigel (BD Biosciences) was polymerized in transwell inserts for 45 min at 37°C. In both assays, cells were plated within the top chamber in medium without serum; the lower chamber, filled with 10% FBS was used as a chemoattractant. Cells were incubated for 24 h and cells that did not migrate or invade through the pores were removed by a cotton swab. Cells on the lower surface of the membrane were stained with crystal violet and counted.

### Animal experiments

To examine the biological importance of HULC in tumorigenesis and metastasis, a nude mouse tumor model was established by subcutaneously injecting LV-HULC siRNA and LV-siRNA-NC transfected Huh-6 or SMMC-7721 cells. LV-HULC siRNA or LV-siRNA-NC transfected Huh-6 cells (2×10^7^cells in 100 μl) were injected subcutaneously into the flanks of each 5-week-old Balb/c athymic nude mice. Tumor growth was examined weekly for 5 weeks. Then mice were euthanized, necropsies were performed, and tumors were weighed. Tumor volumes were calculated by the following formula: V= πAB^2^/6 (A the largest diameter and B the perpendicular diameter). LV-HULC siRNA or LV-siRNA-NC transfected SMMC7721 cells (2×10^7^cells in 100 μl) were injected subcutaneously into the left liver lobes of each nude mice. 5 weeks after SMMC-7721 cells implantation, the mice were sacrificed for analysis and intrahepatic metastases were examined via H&E staining.

### Real-time PCR

Total RNA was extracted by RNAiso Plus (Takara, Dalian, China). The reaction mixture (20 μl) containing 1 μg of total RNA was reversely transcribed to cDNA by using PrimeScript RT-polymerase (Takara, Dalian, China). Quantitative PCR was performed on the cDNA using specific primers (Sangon, Shanghai, China) for HULC (F:5‘-TCATGATGGAATTGGAGCCTT-3′, R: 5′-CTCTTCCTGGCTTGCAGATTG-3′), miR-200a-3p (5′- AACACTGTCTGGTAACGATGTCGT-3′), ZEB1 (F: 5′- CAGCTTGATACCTGTGAATGGG-3′, R: 5′-TATCTGTGGTGTGGGACT-3′) and GAPDH (F: 5′-CAGCCAGGAGAAATCAAACAG-3′, R: 5′-GACTGAGTACCTGAACCGGC-3′). All reactions were performed using the Applied Biosystems 7000 Sequence Detection System (Applied Biosystems, Foster City, CA, USA). Relative expression levels were calculated as ratios normalized against those of GAPDH. Comparative quantification was determined using the 2^−Δ ΔCt^ method.

### Western blot analysis

Protein samples harvested from HCC cells (Huh-6, BEL-7402 or SMMC-7721) and HCC tissues were lysed with denaturing SDS-PAGE sample buffer using standard methods. Protein lysates were separated by 10% or 12%SDS-PAGE and transferred onto nitrocellulose membranes. The membranes were blocked with TBS containing 5% nonfat milk and then incubated with antibodies (ZEB1, E-Cadherin, N-Cadherin, Vimentin, Snail, β-Catenin, ZO-1 or β-actin) at 4°C overnight. After washing, the membranes were incubated with HRP-conjugated anti-IgG at room temperature for 2 hour. Signal detection was carried out with an ECL system (Millipore, Billerica MA, USA).

### Immunohistochemical staining

HCC tissues samples were fixed in 4% mediosilicic isotonic formaldehyde for 24 h, dehydrated and then embedded in paraffin. Five micrometer-thick sections were cut from each paraffinembedded tissue. HCC cells were harvested and 5×10^4^/mL of cells were seeded onto glass coverslips in 24-well plates. The streptavidin-peroxidase (SP) staining technique was used to detect protein following antigen retrieval by microwave treatment. After blocking endogenous peroxidase activity, specimens were incubated with antibodies (ZEB1, E-Cadherin, N-Cadherin or Ki-67) at 4°C overnight. Specimens were incubated at room temperature for 60 min with the secondary antibody. After being rinsed with PBS, diaminobenzidine (DAB) solution was used.

### Statistical analysis

The SPSS19.0 software was used for general statistical analyses. The significance of differences between groups was performed using Student's t-test, one-way analysis of variance (ANOVA), χ^2^ test, Fisher exact test or Wilcoxon test, as appropriate. Survival rate were calculated by the Kaplan-Meier method with the log-rank test applied for comparison. Survival data were evaluated using univariate and multivariate Cox proportional hazards model. All tests performed were two sided and the criterion for statistical significance was established as P< 0.05.
